# Phase I clinical study of RG7356, an anti-CD44 humanized antibody, in patients with acute myeloid leukemia

**DOI:** 10.18632/oncotarget.8687

**Published:** 2016-04-11

**Authors:** Norbert Vey, Jacques Delaunay, Giovanni Martinelli, Walter Fiedler, Emmanuel Raffoux, Thomas Prebet, Carlos Gomez-Roca, Cristina Papayannidis, Maxim Kebenko, Peter Paschka, Randolph Christen, Ernesto Guarin, Ann-Marie Bröske, Monika Baehner, Michael Brewster, Antje-Christine Walz, Francesca Michielin, Valeria Runza, Valerie Meresse, Christian Recher

**Affiliations:** ^1^ Institut Paoli-Calmettes, Marseille, France; ^2^ Aix Marseille Université, Marseille, France; ^3^ Service d'Hématologie Clinique, Hôpital Hôtel-Dieu, Nantes, France; ^4^ Department of Experimental, Diagnostic and Specialty Medicine, University of Bologna, Bologna, Italy; ^5^ Department of Medicine II, University Medical Center Hamburg-Eppendorf, Hamburg, Germany; ^6^ Hôpital Saint Louis, AP-HP, EA3518 Université Paris VII, Paris, France; ^7^ Institut Universitaire du Cancer de Toulouse Oncopole, Toulouse, France; ^8^ Institut Claudius Regaud, Clinical Research Unit, Toulouse, France; ^9^ Department of Internal Medicine III, University Hospital Ulm, Ulm, Germany; ^10^ Product Development, Safety Risk Management, Roche, Basel, Switzerland; ^11^ Pharma Research & Early Development, Roche Innovation Center Basel, Basel, Switzerland; ^12^ Pharma Research & Early Development, Roche Innovation Center Penzberg, Penzberg, Germany; ^13^ Pharma Research & Early Development, Roche Innovation Centre, Welwyn, UK; ^14^ CHU de Toulouse, Université Toulouse III, Toulouse, France

**Keywords:** RG7356, relapsed/refractory acute myeloid leukemia, anti-CD44 humanized antibody, phase I trial, cell adhesion

## Abstract

RG7356, a recombinant anti-CD44 immunoglobulin G1 humanized monoclonal antibody, inhibits cell adhesion and has been associated with macrophage activation in preclinical models. We report results of a phase I dose-escalation study of RG7356 in relapsed/refractory acute myeloid leukemia (AML).

Eligible patients with refractory AML, relapsed AML after induction chemotherapy, or previously untreated AML not eligible for intensive chemotherapy were enrolled and received intravenous RG7356 at dosages ≤ 2400 mg every other week or ≤ 1200 mg weekly or twice weekly; dose escalation started at 300 mg.

Forty-four patients (median age, 69 years) were enrolled. One dose-limiting toxicity occurred (grade 3 hemolysis exacerbation) after one 1200 mg dose (twice-weekly cohort). The majority of adverse events were mild/moderate. Infusion-related reactions occurred in 64% of patients mainly during cycle 1. Two patients experienced grade 3 drug-induced aseptic meningitis. Pharmacokinetics increased supraproportionally, suggesting a target-mediated drug disposition (TMDD) at ≥ 1200 mg. Two patients achieved complete response with incomplete platelet recovery or partial response, respectively. One patient had stable disease with hematologic improvement.

RG7356 was generally safe and well tolerated. Maximum tolerated dose was not reached, but saturation of TMDD was achieved. The recommended dose for future AML evaluations is 2400 mg every other week.

## INTRODUCTION

Acute myeloid leukemia (AML) is the most common form of acute leukemia in adults, and its incidence increases with age. With intensive chemotherapy regimens, complete remission rates between 70% and 80% can be achieved [1, 2]; however, the majority of patients relapse and prognosis is very poor [3]. Treatment options are even more limited in elderly patients because of the high frequency of chemotherapy-resistant forms of AML [2, 4], combined with the inability of the majority of these patients to tolerate intensive treatments [5]. Thus, there is a need to develop new therapies with more effective mechanisms of action and lower toxicity as compared with conventional chemotherapy.

CD44 is an adhesion molecule expressed on hematopoietic precursors, including long-term culture-initiating cells, colony forming unit–granulocyte macrophages, and leukemic cells [6]. Its main ligand is hyaluronic acid (HA), an extracellular matrix glycosaminoglycan present in the bone marrow (BM) microenvironment. Interactions between CD44 and HA are essential to mediate the cellular adhesion and migration of leukemic stem cells (LSC) to the stroma in the BM [7]. Consequently, administration of anti-CD44 monoclonal antibodies (mAb) was associated with eradication of LSC after serial transplants in immunocompromised murine models of AML [8]. In addition, signal transduction by CD44 regulates many cellular functions, including myeloid differentiation [9].

The investigational drug RG7356 is a recombinant immunoglobulin G1 (IgG1) humanized mAb that specifically binds to the standard region of CD44 near the HA binding domain. By blocking the interaction between CD44 and HA, RG7356 inhibits cell adhesion to HA-coated plates at nanomolar concentrations *in vitro* [10]. *In vitro*, the RG7356-mediated disruption of the tumor microenvironment triggers the release of specific chemo-attractants (e.g. CCL2) that recruit and activate macrophages, leading to the phagocytosis of RG7356-opsonized tumor cells (Roche internal data).

Taken together, these data along with the known CD44 biology and its role in leukemia supported the rationale for clinical investigation of RG7356. We report the results of a phase I dose-escalation study of RG7356 in patients with refractory/relapsed AML.

## RESULTS

### Patient characteristics

Forty-four patients were evaluable (Table 1). Half of the patients were ≥ 69 years of age. Thirty-seven patients (84%) had refractory or relapsed disease, including 11 (25%) that had a previous transplant. Seven patients (16%) were previously untreated elderly patients unfit for conventional chemotherapy. Twelve out of 43 patients (28%) had unfavorable cytogenetics, and 11 out of 37 (30%) had *FLT3* mutations. All 42 patients with available pretreatment BM biopsy were positive for CD44 expression on leukemic blasts by immunohistochemistry (IHC).

**Table 1:** Patient characteristics

Characteristic	Number of patients (%) *n* = 44
Median age (range), years	69 (20–82)
≤ 60	15 (34)
> 60	29 (66)
Sex (male/female)	26/18
ECOG performance status at screening	
0	18 (41)
1	21 (48)
2	5 (11)
FAB classification	
M0	4
M1–2	22
M4–5	7
M6	3
sAML	7
Undifferentiated AML	1
Cytogenetics	
Number evaluable	43 (98)
Intermediate risk	31 (72)
Normal karyotype	24 (56)
Other	7 (16)
Unfavorable risk	12 (28)
Complex	6 (14)
Other	6 (14)
*FLT3* mutations	11/37 (30)
ITD	6/37 (16)
TKD	5/37 (14)
*NPM1* mutations	8/34 (24)
Status	
Relapsed/refractory after ≥ 2 lines	5 (11)
Relapsed/refractory after 1 line	21 (48)
Post-transplant relapse	11 (25)
Previously untreated elderly	7 (16)
Median interval from diagnosis to study enrollment (range), months	13 (0.9–130)

Abbreviations: AML, acute myeloid leukemia; ECOG, Eastern Cooperative Oncology Group; FAB, French–American–British; ITD, internal tandem duplication; sAML, secondary acute myeloid leukemia; TKD, tyrosine kinase domain.

Patients were classified into favorable, intermediate, or unfavorable risk groups based on cytogenetics and/or molecular abnormalities. Percentages are calculated on number with evaluable cytogenetics.

### Safety and tolerability

RG7356 was investigated at 4 dose levels and 3 schedules (every other week, weekly, or twice weekly) (Table 2). Median treatment duration was 23 days (range, 1–269 days), and 10 patients (23%) were treated for ≥ 60 days.

**Table 2:** Dose escalation, dose-limiting toxicities, and response

Dose	Schedule	Number of patients	Number of DLT-evaluable patients^a^	DLTs	Response
300 mg	q2w	4	3	0	0
600 mg	q2w	5	3	0	0
1200 mg	q2w	7	4	0	1 CRp, 1 PR
2400 mg	q2w	5	5	0	0
1200 mg	Weekly	9	3	0	0
600 mg	Twice weekly	4	3	0	1 HI
1200 mg^b^	Twice weekly	10	5	1	0

Abbreviations: CRp, complete response with incomplete platelet recovery; DLT, dose-limiting toxicity; HI, hematologic improvement; MTD, maximum tolerated dose; PR, partial response; PK, pharmacokinetics.

a Evaluable patient is defined as any treated patient who previously had a DLT and/or completed the DLT period without having a subsequent DLT.

b At study termination, only one DLT occurred in this cohort out of the 5 evaluable patients; no additional patients were included and the MTD was not determined.

Eighteen patients were not evaluable for dose-limiting toxicity (DLT) determination, owing to disease progression prior to day 21 (*n* = 9), early event of aseptic meningitis (*n* = 2), infusion-related reactions (IRRs) (*n* = 1), incomplete dose on day 1 (*n* = 2), not allowed concomitant medication (*n* = 1), early death due to unrelated fatal pulmonary infection event (*n* = 1), and withdrawn consent (*n* = 2). Only 1 DLT was observed—a grade 3 hemolysis exacerbation occurring after 1 dose of 1200 mg twice weekly. This patient had AML secondary to myelodysplastic syndrome (MDS) with a history of long-lasting red blood cell (RBC) transfusion dependence. Transfusion needs had increased regularly in the weeks preceding RG7356 treatment, concurrently with several episodes of transient increase of unconjugated bilirubin and appearance of alloreactive anti-RhD antibodies. The baseline hemoglobin level was 9.5 g/dL, total bilirubin was 42.3 μmol/L, and lactate dehydrogenase (LDH) was 134 IU/L. One hour after the end of RG7356 administration, the patient presented with unconjugated bilirubin increase (80.4 μmol/L) and severe anemia (hemoglobin level, 6.8 g/dL), although LDH level remained unchanged (137 IU/L). Direct and indirect Coombs tests were positive, and no other markers of hemolysis were present. Although not confirmed, a relationship to RG7356 could not be ruled out.

Because CD44 is normally expressed on human erythrocytes [11], we performed a systematic assessment of direct and indirect Coombs tests in 23 patients. All patients were closely observed for signs of hemolysis. Indirect Coombs test was negative at baseline in all patients for which a screening or pre-dose value was obtained (*n* = 20) and was positive in 15 out of 18 patients (83%) tested after the infusion of cycle 1. Direct Coombs test was positive at baseline in 5 out of 21 patients (24%), and was positive in 16 out of 18 patients (89%) tested after the infusion of cycle 1. No other cases of hemolysis were recorded, including in patients with positive Coombs tests. No evidence of increase in transfusion dependency was observed during the study and across the different cohorts.

The majority of treatment-related adverse events (AEs) were transient and mild to moderate in severity (Table 3). The most frequent treatment-related AEs were grade 1/2 IRRs, the majority of which occurred during the first infusion (59%) compared with subsequent cycles (16%). Incidence of IRRs decreased from 75% to 54% after the slower infusion rate was implemented. There was no apparent correlation between dose, schedule, and incidence or severity of IRRs.

**Table 3:** Drug-related AEs

	Number of patients (%) *n* = 44	
Drug-related event	Any grade	Grade ≥ 3
Any	39 (89)	10 (23)
Total number of AEs	151	13
Infusion-related reactions	28 (64)	0
Pyrexia	14 (32)	0
Headache	7 (16)	0
Coombs indirect test positive	4 (9)	0
Asthenia	4 (9)	2 (5)
Nausea	4 (9)	0
Increased alanine aminotransferase	3 (7)	2 (5)
Vomiting	3 (7)	0
Abdominal pain	2 (5)	0
Increased blood bilirubin	2 (5)	1 (2)
Decreased appetite	2 (5)	0
Fatigue	2 (5)	0
Constipation	2 (5)	0
Rash	2 (5)	0
Aseptic meningitis	2 (5)	2 (5)

Abbreviation: AE, adverse event.

Included are drug-related AEs that occurred in at least 2 patients. The other related AEs of grade ≥3 were increased blood lactate dehydrogenase, febrile neutropenia, hemolysis, neutropenia, tumor lysis syndrome, and decreased white blood cell count experienced by 1 patient each (2%).

Two cases of drug-induced aseptic meningitis (DIAM) were reported early after a dosage of 1200 mg weekly on cycle 1, day 3. Peak concentration (C_max_) in both patients was below the mean C_max_ (369 μg/ml) of the highest evaluated safe dose level (2400 mg), and increases in blood cytokines were in the low range observed for other patients, indicating no dose relationship. In 1 patient, cerebrospinal fluid (CSF) concentration was 0.8% of concomitant RG7356 serum concentration. Although no baseline comparison was possible, we observed high CSF levels of interleukin-10 (IL-10), IL-1 receptor antagonist, macrophage inflammatory protein-1 alpha, macrophage inflammatory protein-1 beta (MIP-1α, MIP-1β), IL-6, IL-8, and macrophage colony stimulating factor-1 in this patient that were consistent with DIAM. Both patients recovered spontaneously within approximately 1 week, but were not rechallenged. No additional cases of DIAM and/or associated neurologic symptoms were recorded after intensification of steroid premedication (methylprednisolone 100 mg was replaced by dexamethasone 20 mg). Maximum tolerated dose (MTD) was not reached for doses escalated up to 2400 mg every other week.

### Pharmacokinetics of RG7356

**Figure 1:**
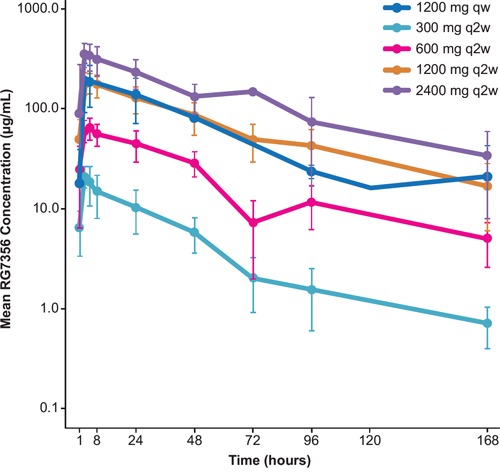
Mean RG7356 concentration for cycle 1 Abbreviations: qw, weekly; q2w, every 2 weeks.

The pharmacokinetic (PK) data for cycle 1 demonstrated that the time to peak concentration (t_max_) occurred shortly after the end of the infusion (3–6 hours) in all cohorts (Supplementary Table 1; Figure 1). For the every-other-week regimen, there was a supraproportional increase in mean exposure (C_max_ and area under the curve [AUC]) from the 300-mg to 1200-mg dose and less than dose proportional from 1200-mg to 2400-mg dose. Total clearance (Cl) and volume of distribution (V_d_) were high (relative to other IgG antibodies) at 300 mg, declined with increasing dose, and plateaued at 1200 mg, at which point target-mediated drug disposition (TMDD) saturation occurred. A similar PK profile was observed following the weekly regimen at the same dose. Mean half-life (t_½_) was 2–3 days and remained the same over the entire dose range.

### Responses

**Figure 2:**
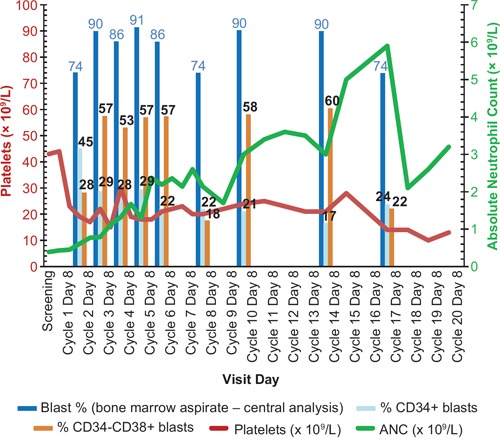
Leukemic stem cell (LSC) differentiation during treatment with RG7356 LSC differentiation during treatment with RG7356 (patient 3015, 600 mg, twice weekly dose regimen, on treatment for 26 cycles). LSC differentiation is shown by reduction of percentage of CD34^+^ blasts and percentage increase of CD34^−^/CD38^+^ blasts in the bone marrow. Hematologic improvement (HI) is shown by absolute neutrophil count (ANC) increase (green line).

One complete response (CR) with incomplete platelet recovery (CRp) and 1 partial response (PR) were recorded. The responders had received RG7356 1200 mg every other day, had normal cytogenetics, and no *FLT3* or *NPM1* mutations. One patient (CRp) was in second relapse following chemotherapy, while the other patient (PR) had previously untreated AML secondary to MDS. Durations of response were 81 and 154 days, respectively. One additional patient in the 600-mg every-other-day dose achieved stable disease with hematologic improvement (HI; neutrophil improvement according to 2006 International Working Group (IWG) Response Criteria in Myelodysplasia [12]) that lasted for 26 cycles (Figure 2). Disease control rate (CR + PR + HI) was 7%. The majority of patients progressed, including 25% who progressed during the first 2 cycles.

### Pharmacodynamics

**Figure 3:**
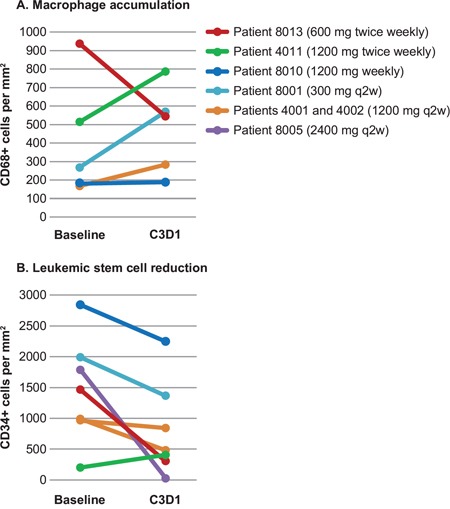
Macrophage and leukemic stem cell changes during treatment with RG7356 **A.** Macrophage (CD68) frequency and **B.** leukemic stem cell (CD34) reduction in the bone marrow was shown by immunohistochemistry analysis at baseline (pre-dose cycle 1, day 1 [C1D1]) and on treatment (pre-dose cycle 3, day 1 [C3D1]).

During treatment with RG7356, we observed a trend for increase of macrophages (CD68^+^) and decrease of stem cell-like AML blasts (CD34^+^) in BM biopsies (Figure 3), in agreement with preclinical data that suggested that the mechanism of action of RG7356 involves active macrophage recruitment and subsequent phagocytic activity against tumor cells (Roche internal data). There were no changes in the CD44 and HA expression pattern in BM (data not shown). One patient with stable disease and HI showed a decrease in CD34^+^ cells and an increase in CD34^−^/CD38^+^ cells, suggesting blast differentiation (Figure 2).

## DISCUSSION

Increased expression of different LSC markers has been associated with poor clinical outcome in patients with AML treated with conventional chemotherapy [13], and therefore, several of those cell surface antigens might serve as therapeutic targets, such as CD123, C-type lectin-like molecule 1 (CLL-1), CD47, T cell Ig mucin 3 (TIM-3), CD96, CXCR4, or CD33 [14-20]. For most of these targets, mAbs have indeed been developed but are still in preclinical or early clinical development. CD44 is a particularly attractive target in AML because its expression on leukemic blasts has been confirmed in 100% of 131 patients with various types of AML (F. Hoffmann-La Roche Ltd, unpublished data). Furthermore, CD44 activation is capable of enhancing AML blast cell survival and resistance to apoptosis [21]. CD44 also plays a key role in LSC homing in the BM, and thus, disrupting the LSC niche is a promising therapeutic approach given the role played by these cells in the resistance to conventional chemotherapy [22]. Our preclinical data show that RG7356 can induce macrophage-mediated phagocytosis of target cells *in vitro*, which may account for most of its direct antitumor activity (Roche internal data).

In this study, we have reported the first results of the administration of RG7356, an IgG1 anti-CD44 mAb, in patients with AML. Increased macrophage frequencies were observed on BM biopsy specimens, as measured by CD68 staining by IHC. Interestingly, this increase in macrophages was paralleled by a decreased frequency of CD34^+^ cells. In addition to this effect, 1 patient who achieved an HI had increased frequencies of CD34^−^/CD38^+^ cells together with a reduction in CD34^+^ cells, suggesting the induction of blast differentiation. This observation is consistent with previous *in vitro* studies that showed that anti-CD44 antibodies may trigger differentiation [23, 24].

The PK analysis indicated that the TMDD was more pronounced in patients with AML (≥ 1200 mg) compared with that observed in patients with solid tumors (≥ 450 mg) (Roche internal data). The PK differed from that expected for an IgG1 monoclonal antibody, with a large V_d_, high total Cl, and short t_½_ of < 4 days, justifying the investigation of biweekly administration.

Overall, RG7356 administration was safe and well tolerated. Only 1 DLT was observed, and MTD was not reached at dosages up to 2400 mg every other week. The most frequent AEs were moderate IRRs, which decreased substantially after premedication intensification. Indirect Coombs tests’ positivity was observed in 83% of the tested patients following RG7356 administration; however, this was not associated with hemolysis, except in 1 patient who presented with hemolysis exacerbation after the first infusion. In toxicology studies (*in vitro* blood compatibility studies), RG7356 did not show any hemolytic potential (F. Hoffmann-La Roche Ltd, unpublished data). Based on these data, the risk of hemolysis following RG7356 administration seems minimal, but the occurrence of false-positive immunohematologic tests has to be anticipated for patients who may require RBC transfusions. This is explained by the high expression of CD44 on erythrocytes [25], which carries the Indian blood group system [26].

Two cases of DIAM were observed, both following the first infusion of RG7356. Recovery was achieved within a few days following steroid administration. As a consequence, premedication using dexamethasone was recommended by the protocol data safety monitoring group, and no additional cases were recorded precluding more extensive biologic analysis of suspected DIAM. DIAM is a rare complication observed following the administration of various drugs, such as nonsteroidal anti-inflammatory drugs, intrathecal chemotherapy, antiepileptic drugs, and immunomodulatory/anti-inflammatory agents, including intravenous Igs or mAbs [27-29]. The pathophysiology of these 2 cases remains elusive, and may include direct interaction of RG7356 with meninges, induction of an inflammatory response, or hypersensitivity reactions. Recently, an increasing number of DIAM cases following the administration of various mAbs has been reported [30]. Overall, cases of DIAM are probably underreported, and attention should be paid to this rare complication in patients treated with mAbs.

Altogether, our data show that the administration of RG7356 doses able to saturate TMDD in a time- and concentration-dependent manner was safe. Limited clinical activity was seen with a disease control rate of 7% in a population of patients with advanced AML; 1 CRp, 1 PR, and 1 stable disease with HI that was associated with reduced CD34^+^ cells were reported. It is worth noting that the goal of an anti-CD44-specific approach in AML is to target the LSC compartment. In the context of this phase I trial, treatment activity was evaluated by the response rate, which was tailored to measure bulk tumor cell killing that did not necessarily reflect the effects on LSC. Indeed, demonstration of an effect on putative LSC in humans remains a challenge using classic response criteria [31].

In conclusion, the administration of RG7356 is safe at a recommended dose of 2400-mg every-other-week, 1200 mg weekly, or 600 mg twice weekly. Based on the observed short t_½_, a more frequent administration schedule (e.g. 3 times a week) might also be worth testing. The limited clinical activity seen here does not support the use of RG7356 as monotherapy on patients with florid AML; however, investigation in the context of minimal residual disease might represent a means to unravel selective effects on LSC. Furthermore, the lack of clinical activity but favorable toxicity profile of RG7356 as a monotherapy support the rationale for further investigation as a combination therapy with cytotoxic agents such as cytarabine or in another clinical setting, such as consolidation or maintenance therapy.

## MATERIALS AND METHODS

### Patient selection

Key inclusion/exclusion criteria are provided in the Supplemental Material. Briefly, patients were eligible if they were diagnosed with AML according to the World Health Organization's criteria, and provided signed written informed consent.

Patients were eligible regardless of CD44 expression. CD44 expression had previously been evaluated by IHC staining in a series of 131 trephine BM biopsies from patients with AML of all subtypes and stages of disease; all samples were CD44^+^ (F. Hoffmann-La Roche Ltd, unpublished data). Patients were classified into favorable, intermediate, and unfavorable risk groups on the basis of cytogenetics and/or molecular abnormalities [32].

### Study design

The study was conducted in 8 centers in France, Italy, and Germany following approval by each country's Institutional Review Board and registration on ClinicalTrials.gov (study identifier: NCT01641250), and was performed in accordance with the principles of the Declaration of Helsinki. Multiple doses and schedules of RG7356 were assessed with the aim of determining the MTD/optimal biologic dose (OBD). A standard “3 + 3” design was used. Cohorts of at least 3 patients were enrolled in one of the RG7356 dosage levels administered consecutively on an every other week, weekly, or twice weekly schedule for each 14-day cycle.

The DLT period was defined as the first 21 study days following first administration. The definition of DLT is available in the Supplemental Material. Hypersensitivity reactions and IRRs were not considered dose-limiting.

In the absence of DLT, the RG7356 dose was escalated by 100% increments until MTD or OBD was reached. MTD was defined as the highest dose level below which at least 2 patients in a dose cohort experienced a DLT. The OBD was defined as the dose that demonstrated the maximum/optimal pharmacodynamic activity and PK properties.

### Treatment

Based on the results of the phase I study in patients with CD44^+^ solid tumors (NCT01358903) (Roche internal data), the starting dose was defined as 300 mg every other day. Flat doses were used. The RG7356 treatment schedule is provided in the Supplemental Material. Premedication consisting of acetaminophen, diphenhydramine, and corticoids (methylprednisolone replaced by dexamethasone in the course of the study) was mandatory for the first 2 infusions.

### Response and toxicity assessments

BM aspirates were performed every 14 days provided that the patient remained in the study. IWG response criteria were used [33]. For patients showing no response, HI was defined as cytopenia improvements according to the 2006 IWG Response Criteria in Myelodysplasia [12]. All patients who received at least 1 dose of RG7356 were included for general safety or efficacy evaluations. Toxicity was evaluated using the National Cancer Institute Common Terminology Criteria for Adverse Events version 4.03.

### Pharmacokinetic assessments

Samples were taken during cycle 1 on days 1, 2, 3, and 8 and cycle 2 on day 1. Estimation of the PK parameters were performed using standard noncompartmental (model independent) methods in cycle 1. Actual sampling time was used to calculate PK parameters. In all calculations, 0 was substituted for concentrations below the quantification limit of the assay. Total clearance, V_d_, t_1/2_, t_max_, C_max_, AUC, individual and mean serum RG7356 concentrations versus time, and interpatient variability were assessed. To determine the influence of antigen expression on actual drug distribution and/or elimination, the TMDD was determined. The PK analysis was performed using Phoenix WinNonLin version 6.2.

### Pharmacodynamic assessments

Whole blood was sampled to assess routine blast cell counts, circulating CD44^+^ leukemic blasts, and stem cell population by multicolor flow cytometry. In addition to routine AML markers on blasts, immunophenotyping included the assessment of circulating LSC (CD44, CD34, CD38, and/or additional markers), T cells (CD3, CD4, CD8), B cells (CD19), NK cells (CD3, CD16/56), and monocytes (CD14), and their respective CD44 expression levels.

Serial BM aspirates were collected for morphological routine assessments, CD44-expressing blast cell counts, and stem cell population assessment by multicolor flow cytometry. BM biopsy samples were collected at pre-dose cycle 1, pre-dose cycle 3, and at progression for routine assessments (cellularity, percent blasts) and for exploratory markers including CD44, HA, CD68, and CD34 assessment by IHC.

## SUPPLEMENTARY MATERIALS AND METHODS TABLE


