# Supplementary material for: Phase I clinical study of RG7356, an anti-CD44 humanized antibody, in patients with acute myeloid leukemia
Source: Oncotarget. 2016 Apr 11;7(22):32532–42. doi: 10.18632/oncotarget.8687 (PMC5078031; doi:10.18632/oncotarget.8687)
Supplement: Supplementary file 1 [file oncotarget-07-32532-s001.pdf]

Phase I clinical study of RG7356, an anti-CD44 humanized antibody, in patients with acute myeloid leukemia

SUPPLEMENTARY MATERIALS AND METHODS

Key inclusion/exclusion criteria

Eligible patients were aged > 18 years and ≤ 60 years old in second relapse and beyond or refractory in first relapse (i.e. failure of at least 1 cytarabine-containing regimen) or primary refractory after at least 2 induction courses; > 60 years old in relapse and/or failure of at least 1 cytarabine-containing induction regimen; > 70 years old previously untreated and not eligible for conventional induction chemotherapy; relapse following hematopoietic stem cell transplantation; Eastern Cooperative Oncology Group performance status of 0–2; white blood cell count < 30 × 10^9/L; adequate hepatic function (bilirubin ≤ 2 mg/dL, aspartate aminotransferase (AST)/alanine transaminase (ALT) ≤ 2.5 × upper limit of normal or renal function [normal serum creatinine]); and no prior therapy for ≥ 14 days with the exception of hydroxyurea; increased QTc interval (QTc > 470 ms).

Dose-limiting toxicity (DLT) definition

DLT was defined as: (1) any nonhematologic toxicity grade ≥ 3 attributable to RG7356 except for fatigue, anorexia, and alopecia; (2) grade 3 aspartate AST or ALT toxicity lasting for > 7 days; or (3) prolonged myelosuppression (i.e. marrow hypocellularity without evidence of leukemia [< 5% blasts] on day 42 or later from start of therapy).

Treatment

RG7356 was supplied in 2 pharmaceutical forms as 200-mg vials (Ro 542-9083/F04, cohorts 1–6) or 250-mg vials (Ro 542-9083/F05, cohort 7) by the sponsor (F. Hoffmann-La Roche Ltd, Basel, Switzerland). Initially, the first infusion was administered over 3 hours. From 2400 mg q2w cohort onward, the first infusion started at a rate of 10 mg/h for the first hour to reduce the severity of IRRs. Thereafter, the infusion rate was escalated in 30-minute intervals up to a maximum infusion rate of 800 mg/h. If the first infusion was well tolerated, subsequent infusions started at a rate of 20 mg/h for 15 minutes and were escalated in 15-minute intervals up to an infusion rate of 800 mg/h.

RESULTS

Supplemental Table 1: PK for RG7356

Regimen (dose schedule)	Statistic	$t_{½}$ (h)	t_{max}[a] (h)	C_{max} (μg/mL)	AUC (h* μg/mL)	V_d (mL)	Cl (mL/h)
300 mg q2w	*n*	4	4	4	4	4	4
	Mean	63.0	3.29	20.6	750	44600	523
	SD	24.4	0.649	6.17	448	21200	338
	CV%	38.7	18.5	30.0	59.8	47.6	64.6
600 mg q2w	*n*	5	5	5	5	5	5
	Mean	43.8	5.00	62.9	3450	11600	195
	SD	12.2	2.68	16.9	1400	3130	82.8
	CV%	27.8	48.0	26.9	40.5	27.1	42.4
1200 mg q2w	*n*	5	5	5	5	5	5
	Mean	59.2	5.25	185	12400	8880	105
	SD	5.79	1.96	39.1	4150	3050	36.5
	CV%	9.8	35.0	21.1	33.5	34.3	34.8
2400 mg q2w	*n*	5	5	5	5	5	5
	Mean	58.5	6.00	329	21100	12000	136
	SD	11.0	0.729	101	9450	8650	72.7
	CV%	18.8	11.1	30.8	44.8	72.0	53.3
1200 mg weekly	*n*	NC	9	9	9	NC	NC
	Mean	NC	6.62	175	9980	NC	NC
	SD	NC	1.95	79.2	4490	NC	NC
	CV%	NC	30.4	45.1	45.0	NC	NC

Abbreviations: AUC, area under the curve; C_{max}, peak concentration; Cl, clearance; CV, coefficient of variation; NC, not collected; PK, pharmacokinetics; SD, standard deviation; $t_{½}$, half-life; t_{max}, time to peak concentration.
[a]Median.
